# Longitudinal Clinical and Cognitive Changes Along the Alzheimer Disease Continuum in Down Syndrome

**DOI:** 10.1001/jamanetworkopen.2022.25573

**Published:** 2022-08-05

**Authors:** Laura Videla, Bessy Benejam, Jordi Pegueroles, María Carmona-Iragui, Concepción Padilla, Susana Fernández, Isabel Barroeta, Miren Altuna, Silvia Valldeneu, Diana Garzón, Laia Ribas, Víctor Montal, Javier Arranz Martínez, Mateus Rozalem Aranha, Daniel Alcolea, Alexandre Bejanin, Maria Florencia Iulita, Sebastià Videla Cés, Rafael Blesa, Alberto Lleó, Juan Fortea

**Affiliations:** 1 Barcelona Down Medical Center, Fundació Catalana Síndrome de Down, Barcelona, Spain; 2 Sant Pau Memory Unit, Department of Neurology, Institut d’Investigacions Biomèdiques Sant Pau–Hospital de Sant Pau, Universitat Autònoma de Barcelona, Barcelona, Spain; 3 Centro de Investigación Biomédica en Red en Enfermedades Neurodegenerativas (CIBERNED), Madrid, Spain; 4 Clinical Research Support Unit, Bellvitge Biomedical Research Institute, Department of Clinical Pharmacology, University of Barcelona, Barcelona, Spain

## Abstract

**Importance:**

Alzheimer disease (AD) is the main medical problem in adults with Down syndrome (DS). However, the associations of age, intellectual disability (ID), and clinical status with progression and longitudinal cognitive decline have not been established.

**Objective:**

To examine clinical progression along the AD continuum and its related cognitive decline and to explore the presence of practice effects and floor effects with repeated assessments.

**Design, Setting, and Participants:**

This is a single-center cohort study of adults (aged >18 years) with DS with different ID levels and at least 6 months of follow-up between November 2012 and December 2021. The data are from a population-based health plan designed to screen for AD in adults with DS in Catalonia, Spain. Individuals were classified as being asymptomatic, having prodromal AD, or having AD dementia.

**Exposures:**

Neurological and neuropsychological assessments.

**Main Outcomes and Measures:**

The main outcome was clinical change along the AD continuum. Cognitive decline was measured by the Cambridge Cognitive Examination for Older Adults With Down Syndrome and the modified Cued Recall Test.

**Results:**

A total of 632 adults with DS (mean [SD] age, 42.6 [11.4] years; 292 women [46.2%]) with 2847 evaluations (mean [SD] follow-up, 28.8 [18.7] months) were assessed. At baseline, there were 436 asymptomatic individuals, 69 patients with prodromal AD, and 127 with AD dementia. After 5 years of follow-up, 17.1% (95% CI, 12.5%-21.5%) of asymptomatic individuals progressed to symptomatic AD in an age-dependent manner (0.6% [95% CI, 0%-1.8%] for age <40 years; 21.1% [95% CI, 8.0%-32.5%] for age 40-44 years; 41.4% [95% CI, 23.1%-55.3%] for age 45-49 years; 57.5% [95% CI, 38.2%-70.8%] for age ≥50 years; *P* < .001), and 94.1% (95% CI, 84.6%-98.0%) of patients with prodromal AD progressed to dementia with no age dependency. Cognitive decline in the older individuals was most common among those who progressed to symptomatic AD and symptomatic individuals themselves. Importantly, individuals with mild and moderate ID had no differences in longitudinal cognitive decline despite having different performance at baseline. This study also found practice and floor effects, which obscured the assessment of longitudinal cognitive decline.

**Conclusions and Relevance:**

This study found an association between the development of symptomatic AD and a high risk of progressive cognitive decline among patients with DS. These results support the need for population health plans to screen for AD-related cognitive decline from the fourth decade of life and provide important longitudinal data to inform clinical trials in adults with DS to prevent AD.

## Key Points

**Question:**

Are age, intellectual disability (ID), and clinical status associated with Alzheimer disease (AD) progression and longitudinal cognitive decline in adults with Down syndrome (DS)?

**Findings:**

In this cohort study of 632 adults with DS, there was a high age-dependent risk of developing symptomatic AD but not the prodromal stage of the disease. ID stratification was not associated with longitudinal cognitive decline, and the study found both practice and floor effects.

**Meaning:**

These findings show the need for health plans to screen for AD in adults with DS and provide important data to inform AD clinical trials.

## Teaser

This cohort study examines clinical progression along the Alzheimer disease continuum and its related cognitive decline among adults with Down syndrome.

## Introduction

Down syndrome (DS) is the most frequent cause of intellectual disability (ID) of genetic origin, affecting 5.8 million people worldwide.^1^ In adults with DS, Alzheimer disease (AD) is the main medical problem and main cause of death.^2^ Indeed, the AD pathological hallmarks are universal by age 40 years,^3^ and the dementia prevalence increases exponentially thereafter,^4,5,6,7^ with a cumulative incidence of more than 95% in the seventh decade. This is mainly owing to the presence of an extra copy of the amyloid-β precursor protein gene, which is coded in chromosome 21.^8^ Consequently, DS is considered a genetic form of dementia, similar to autosomal dominant AD (ADAD).^2,6,9^ Importantly, the clinical and AD biomarker changes are strikingly similar in both populations.^6^

ID is defined as a condition characterized by substantial limitations in intellectual functioning, as well as in adaptive behavior. The premorbid ID associated with DS can overshadow AD-related cognitive decline, and it also explains the floor effects found in traditional neuropsychological tests used in general population. Furthermore, health professionals from the general population do not feel confident when attending people with DS.^10^ For these reasons, people with DS require adapted tests to assess cognitive performance, as well as specific medical care.^11^ Recent studies^12^ show that adapted neuropsychological tests are useful for the diagnosis of prodromal and AD dementia at a cross-section when stratifying by the level of ID. Some tests, such as the modified Cued Recall Test (mCRT), are also useful to capture early AD-associated cognitive decline in asymptomatic adults with DS.^13,14^ However, given differences in premorbid ID level, clinical guidelines have emphasized the need for tracking within-person changes over time to detect AD-related cognitive decline.^15^ There are, however, only a few studies^16,17,18,19^ that have assessed longitudinal AD-related cognitive decline. These studies^2,20,21^ have shown early declines in episodic memory and executive function, but most of them had a small sample size and/or short duration of follow-up, and none of them stratified the findings by ID or age ranges. Finally, floor effects and practice effects can obscure the measurement of cognitive decline, thus affecting cognitive end points in AD clinical trials in this population.^22^ These effects have not been assessed in the AD continuum in DS.

This study evaluated the largest single-center, population-based longitudinal cohort of adults with DS to examine the clinical and the cognitive changes along the AD continuum. We also explored the presence of practice and floor effects.

## Methods

### Study Design, Setting, and Population

This is a single-center, prospective, longitudinal, cohort study of adults with DS recruited at the Alzheimer-Down Unit from the Catalan Down Syndrome Foundation and Hospital of Sant Pau, in Barcelona, Spain. We recruited participants of both sexes aged 18 years or older from a population-based health plan designed to screen for AD in adults with DS in Catalonia. This health plan includes structured semiannual or annual neurological and neuropsychological assessments by experienced clinicians. We included individuals with all levels of ID and a minimum follow-up of 6 months. Individuals with severe and profound ID were excluded in the cognitive analyses, as these individuals perform at floor scores.^12^ eFigure 1 in the Supplement shows the study flowchart.

The study was approved by the Sant Pau Research Ethics Committees, following the standards for medical research in humans recommended by the Declaration of Helsinki.^23^ All participants or their legally authorized representative gave written informed consent. Confidentiality was guaranteed in accordance with current Spanish legislation. This report follows the Strengthening the Reporting of Observational Studies in Epidemiology (STROBE) reporting guideline.

### Study Outcomes

The study procedures included a medical examination with the participant and main caregiver, as well as a neuropsychological assessment whenever possible.^12,24^ For further details of the diagnostic process, see the eAppendix in the Supplement.

The neuropsychological assessment included the Cambridge Cognitive Examination for Older Adults With Down Syndrome (CAMCOG-DS) Spanish version^25^ and the mCRT.^13^ ID was categorized as mild, moderate, severe, or profound according to the *Diagnostic and Statistical Manual of Mental Disorders, Fifth Edition*, and on the basis of caregivers’ reports of the individuals’ best-ever level of functioning and the score of the Kaufman Brief Intelligence Test Spanish version.^26^

The CAMCOG-DS is an adapted cognitive battery with a maximum score of 109. The mCRT is an adapted test to assess free and cued episodic memory, and its maximum score is 36.^13^ In the main text we show the free immediate recall (FIR) score. In both tests, higher scores indicate better cognition. We defined practice effects as any change or improvement that results from repetition of task items, and floor effects as the situation in which a large proportion of participants perform very poorly on a task.^27^

Participants were classified clinically into 4 groups in a consensus meeting between the neurologist and neuropsychologist after independent visits: (1) asymptomatic (ie, no clinical or neuropsychological suspicion of AD), (2) prodromal AD (ie, suspicion of AD, but symptoms did not fulfill criteria for dementia), (3) AD dementia (ie, full-blown AD dementia), and (4) uncertain or nondegenerative neurocognitive disorder (ie, when there were medical, pharmacological, or psychiatric condition interfering with cognition or daily living activities, but no suspicion of neurodegenerative origin). Of note, in some instances, these conditions were treatable and reversible, and individuals were classified in 1 of the other 3 categories at follow-up visits. We excluded all the visits with an uncertain diagnosis. For the prognostic evaluation, asymptomatic participants and those with prodromal AD were subsequently classified as progressors when there was a change in the clinical diagnosis along the AD continuum. Participants who remained in the same AD diagnostic category were classified as nonprogressors.

To estimate longitudinal cognitive decline in the different clinical groups, we included all data points from baseline for each category. For prodromal AD and AD dementia, we also included the data points of progressors after the change in diagnostic category.

### Statistical Analysis

To assess the descriptive statistics for the baseline data, we performed analysis of variance tests for numerical variables and χ^2^ tests of independence for categorical variables. Analyses were performed in R statistical software version 3.6.3 (R Project for Statistical Computing).

To assess clinical progression, we used Kaplan-Meier curves in the whole sample and in different age ranges, in the latter followed by log-rank tests. We used linear mixed-effects models (LME) in the R lme4 package to model the longitudinal cognitive changes as a function of age in individuals with mild and moderate ID separately, including linear and quadratic (when significant) age terms as fixed effects and participant-specific intercepts and slopes as random factors. We tested the interaction term between clinical diagnosis and time. Both raw cognitive scores and cognitive annualized change (follow-up – baseline / years between both time points) were used separately as dependent variables in these analyses. We also assessed the longitudinal cognitive decline in each clinical diagnostic group by applying an LME with an interaction term between diagnostic group and years of follow-up using participant-specific intercepts and slopes as random factors. We finally divided the sample into age ranges and applied an LME with an interaction term between the age intervals and years of follow-up with random intercept and slope for each individual. To assess the practice and floor effects, we plotted the mean cognitive scores at each year of follow-up (for each age range and the different clinical groups, respectively). For the latter, we also modeled the annualized change with respect to the baseline performance with generalized additive models calculated with the R mgcv package with random effects (intercept and slope) for each participant. All statistical analyses were performed using 2-sided tests with a level of significance at *P* < .05.

## Results

### Study Population

eFigure 1 in the Supplement shows the study flowchart. From November 2012 to December 2021, we included 632 adults with DS (mean [SD] age, 42.6 [11.4] years; 292 women [46.2%]) who had longitudinal clinical follow-up visits. Of these, 433 (68.5%) had longitudinal neuropsychological assessments. The Table displays baseline demographic and cognitive data by clinical diagnosis for the whole sample and for the subgroup with longitudinal cognitive assessments: 436 individuals (69.0%) were asymptomatic, 69 (10.9%) had prodromal AD, and 127 (20.1%) had AD dementia. As expected, asymptomatic individuals were younger and had higher cognitive scores than patients with prodromal AD and AD dementia (Table). There were no significant differences in sex distribution, but there were differences in ID across the clinical groups; there was a higher proportion of individuals with moderate ID in all groups. The mean (SD) follow-up in the whole cohort was 28.8 (18.7) months. The follow-up interval was longer in asymptomatic individuals (mean [SD], 31.0 [18.8] months) than in those with prodromal AD (mean [SD], 18.2 [18.8] months) or those with AD dementia (mean [SD], 26.9 [16.2] months) (Table).

**Table.** Demographic and Cognitive Variables by Clinical Diagnosis at Baseline for the Whole Sample and the Cognitive Analysis Subsample

Characteristic	Patients, No. (%)			*P* value
	Asymptomatic	Prodromal AD	AD dementia	
Whole sample (n = 632)				
No.	436	69	127	NA
Sex				
Female	199 (45.6)	32 (46.4)	61 (48.0)	NA
Male	237 (54.4)	37 (53.6)	66 (52.0)	
Age, mean (SD), y	38.0 (10.4)	50.9 (4.9)	53.7 (5.6)	<.001
Intellectual disability				
Mild	101 (23.2)	10 (14.5)	10 (7.9)	<.001
Moderate	206 (47.2)	46 (66.6)	67 (52.8)	
Severe to profound	129 (29.6)	13 (18.8)	50 (39.4)	
Follow-up duration, mo				
Mean (SD)	31.0 (18.8)	18.2 (18.8)	26.9 (16.2)	<.001
Mean (IQR)	29.5 (3.4-60.0)	29.5 (0.7-57.9)	25.8 (1.7-59.6)	
Main drugs				
Antiepileptic	17 (3.9)	4 (5.8)	34 (26.8)	NA
Cholinesterase inhibitors	1 (0.2)	0	3 (2.4)	
Antidepressant	36 (8.3)	4 (5.8)	34 (26.8)	
Antipsychotic	41 (9.4)	4 (5.8)	31 (24.4)	
Cognition sample (n = 433)				
No.	304	53	76	NA
Sex				
Female	149 (49.0)	26 (49.1)	40 (52.6)	NA
Male	155 (51.0)	27 (50.9)	36 (47.4)	
Age, mean (SD)	36.9 (9.6)	51.0 (5.1)	53.0 (5.6)	<.001
Intellectual disability				
Mild	100 (22.9)	10 (18.9)	10 (13.2)	<.001
Moderate	204 (67.1)	43 (81.1)	66 (88.8)	
CAMCOG-DS score, mean (SE)	75.6 (16.0)	47.5 (18.7)	47.1 (17.1)	<.001
mCRT FIR score, mean (SE)	19.5 (6.17)	8.82 (5.93)	4.09 (4.57)	<.001
Follow-up duration, mo				
Mean (SD)	32.5 (18.5)	18.3 (19.2)	27.0 (15.8)	<.001
Mean (IQR)	27.0 (5.4-60.0)	29.2 (0.7-57.9)	23.2 (1.7-59.6)	
Main drugs				
Antiepileptic	4 (1.3)	2 (3.8)	19 (25.0)	NA
Cholinesterase inhibitors	1 (0.3)	0	2 (2.6)	
Antidepressant	20 (6.6)	1 (1.9)	19 (25.0)	
Antipsychotic	21 (6.9)	1 (1.9)	15 (19.7)	

Abbreviations: AD, Alzheimer disease; CAMCOG-DS, Cambridge Cognitive Examination for Older Adults With Down Syndrome; mCRT, modified Cued Recall Test; NA, not applicable.

### Clinical Progression

**Figure 1.**
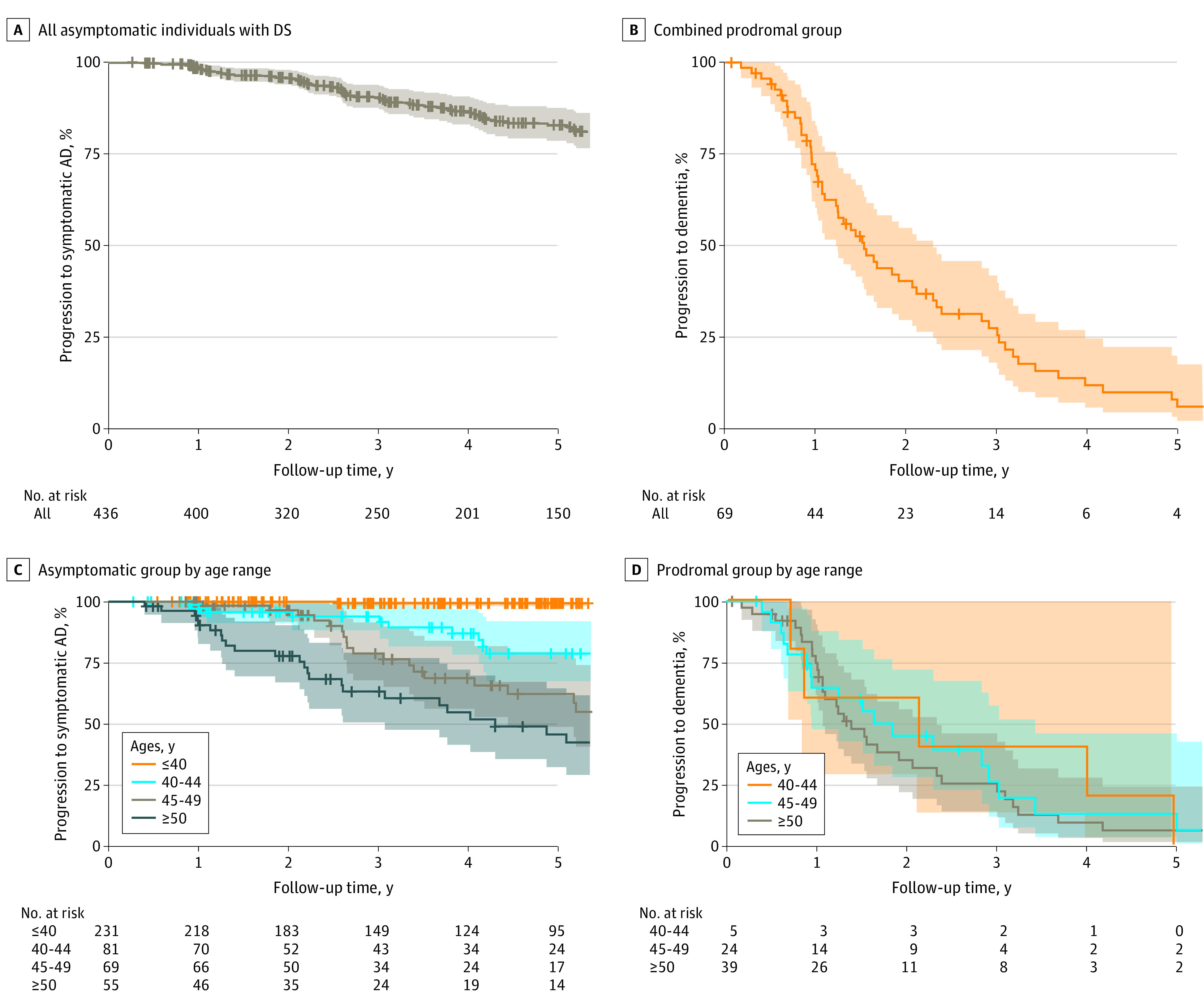
Clinical Progression of Asymptomatic Individuals and Those With Prodromal Alzheimer Disease (AD) Among Adults With Down Syndrome (DS) Kaplan-Meier curves are shown for all asymptomatic individuals (A), all those with prodromal AD (B), asymptomatic individuals by age range (C), and those with prodromal AD by age range (D). Shaded areas indicate 95% CIs.

Figure 1 shows the Kaplan-Meier curves for the clinical progression in the whole sample and for the different age ranges in asymptomatic individuals and those with prodromal AD separately. Overall, after 5 years of follow-up, 17.1% (95% CI, 12.5%-21.5%) of the asymptomatic individuals had progressed to symptomatic AD (Figure 1A), and 94.1% (95% CI, 84.6%-98.0%) of the prodromal group had progressed to dementia (Figure 1B). eTable 1 in the Supplement shows the progression rates at different follow-up times in the different age ranges. The clinical progression in asymptomatic individuals showed a clear age dependency: only 0.6% (95% CI, 0.0%-1.8%) of individuals younger than 40 years in the asymptomatic group progressed to symptomatic AD after 5 years of follow-up, whereas 57.5% (95% CI, 38.2%-70.8%) of those older than 50 years did (corresponding percentages were 21.1% [95% CI, 8.0%-32.5%] for those aged 40-44 years and 41.4% [95% CI, 23.1%-55.3%] for those aged 45-49 years; *P* < .001) (Figure 1C). Progression to AD dementia in patients with prodromal AD, on the other hand, was almost universal after 5 years of follow-up, and, importantly, it did not show such an age dependency. eFigure 2 in the Supplement shows these results stratified by ID (eFigure 2A and 2B in the Supplement) and by sex (eFigure 2C and 2D in the Supplement). There were no significant differences by ID or between men and women in asymptomatic individuals. However, women with prodromal AD had a faster progression than men (χ^2^_1_ = 4.3; *P* = .04).

### Longitudinal Cognitive Outcomes

We next analyzed the longitudinal cognitive data. We first studied the percentage of individuals who were able to complete the cognitive tests at the different follow-up visits by clinical diagnosis and level of ID (eFigure 3 in the Supplement). During the follow-up period, many individuals in symptomatic stages were not able to complete the tests.

**Figure 2.**
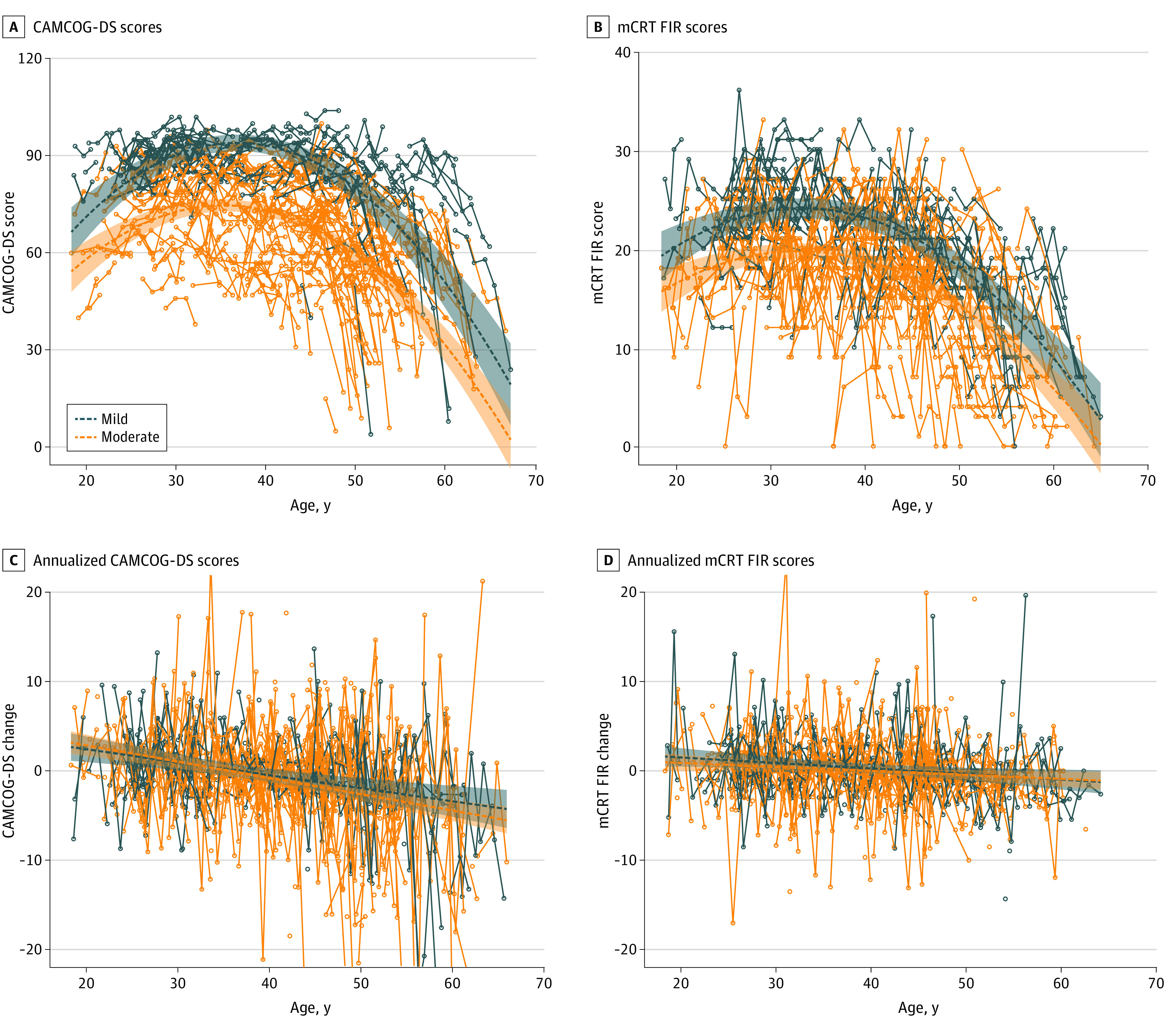
Changes in Cambridge Cognitive Examination for Older Adults With Down Syndrome (CAMCOG-DS) and Modified Cued Recall Test (mCRT) Free Immediate Recall (FIR) Scores With Age in Individuals With Mild and Moderate Levels of Intellectual Disability (ID) Separately Graphs show quadratic association between age and CAMCOG-DS raw scores (A) and mCRT FIR raw scores (B) in patients with mild (blue dots) and moderate (orange dots) ID. Panels C and D show the association between the annualized cognitive change and age by ID in CAMCOG-DS (C) and mCRT FIR (D).

Figure 2 shows the changes in the CAMCOG-DS and mCRT performance with age in adults with DS (individuals with mild and moderate levels of ID are analyzed separately). As expected, the scores were higher for individuals with mild ID than those with moderate ID at all ages (mean [SE] difference, −19.24 [1.68] for CAMCOG-DS and −4.06 [0.55] for mCRT; *P *< .001) (Figures 2A and 2B), but the interaction term of ID with age was not significant, suggesting that there were no differences in the trajectories between the ID groups. Similarly, and importantly, the annualized change did not differ between individuals with mild and moderate ID in either test (Figures 2C and 2D), but showed a similar cognitive decline with age (mean [SE] score difference of −0.15 [0.04] points per year on the CAMCOG-DS and −0.06 [0.02] points per year on the mCRT). Of note, the LME analyses showed a significant age quadratic term in the model for the raw scores for both tests and increases in the longitudinal performance in younger individuals, suggesting the presence of practice effects (see eFigure 4 in the Supplement for CRT total immediate recall results). Sex was not associated with cognitive performance with age, or in the annual change, except for the raw CAMCOG-DS in the mild ID group, where women had a quadratic trajectory different than that of men (β [SE], −360.68 [32.36] for CAMCOG-DS and 85.90 [11.71] for mCRT; *P* = .006), although they did not decline faster than men.

**Figure 3.**
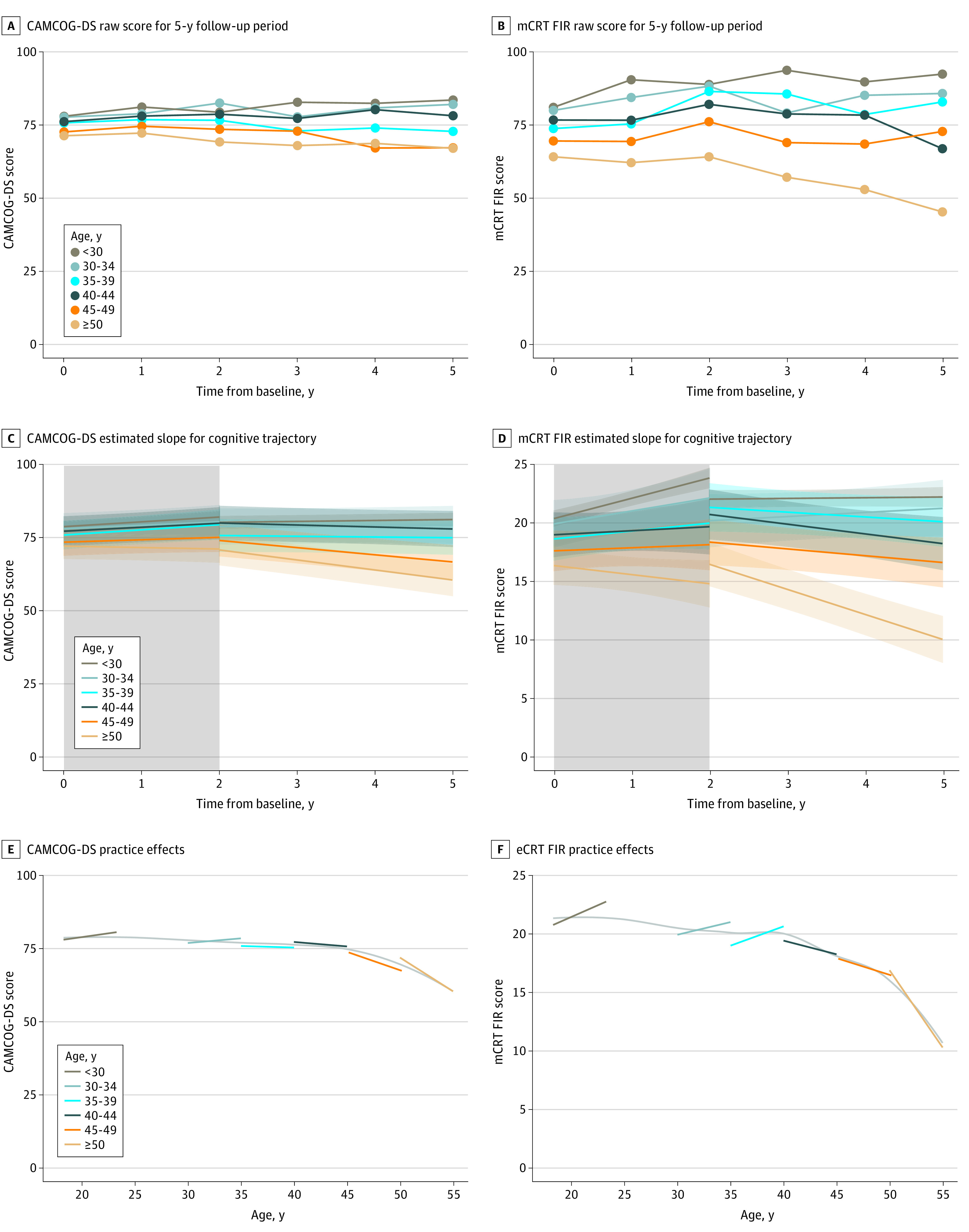
Cognitive Trajectories by Age Ranges in Asymptomatic Participants With Down Syndrome (DS) Showing Learning Effects in Younger Individuals During the First 2 Years of Follow-up Panels A and B show Cambridge Cognitive Examination for Older Adults With Down Syndrome (CAMCOG-DS) (A) and modified Cued Recall Test (mCRT) free immediate recall (FIR) (B) raw scores by age ranges along 5 years of follow-up. Panels C and D show CAMCOG-DS (C) and mCRT FIR (D) estimated slopes for the cognitive trajectories during the first 2 years of follow-up and beyond calculated separately by age ranges during the follow-up. Panels E and F show that CAMCOG-DS (E) and mCRT FIR (F) practice effects were seen several years after a decline in (baseline) cognitive scores with age was observed.

To assess the presence of practice effects, we first analyzed the longitudinal cognitive trajectories in the asymptomatic individuals in the different age ranges. These analyses showed the presence of practice effects during the first 2 years in asymptomatic individuals (mainly in the mCRT FIR), either as early increases in the cognitive tests with subsequent stabilization, or as early stability with subsequent decline (Figures 3A and 3B). We, therefore, estimated the trajectories for the CAMCOG-DS and mCRT FIR scores for the first 2 years of follow-up and those beyond separately (Figures 3C and 3D) to estimate the cognitive decline when there are no practice effects (those after 2 years of follow-up). We finally estimated the longitudinal trajectory of change for the 2 tests in the different age ranges (Figures 3E and 3F). Practice effects (apparent as longitudinal improvement in the cognitive tests) were clear in younger individuals (see eFigure 5 in the Supplement for CRT total immediate recall results). In the stratified analyses by sex, there were no significant differences, except for the trajectory in CAMCOG-DS after 2 years of follow-up in the age group of 40 to 49 years, where women declined faster than men (mean [SE], 2.90 [1.36] points per year; *P* = .04).

**Figure 4.**
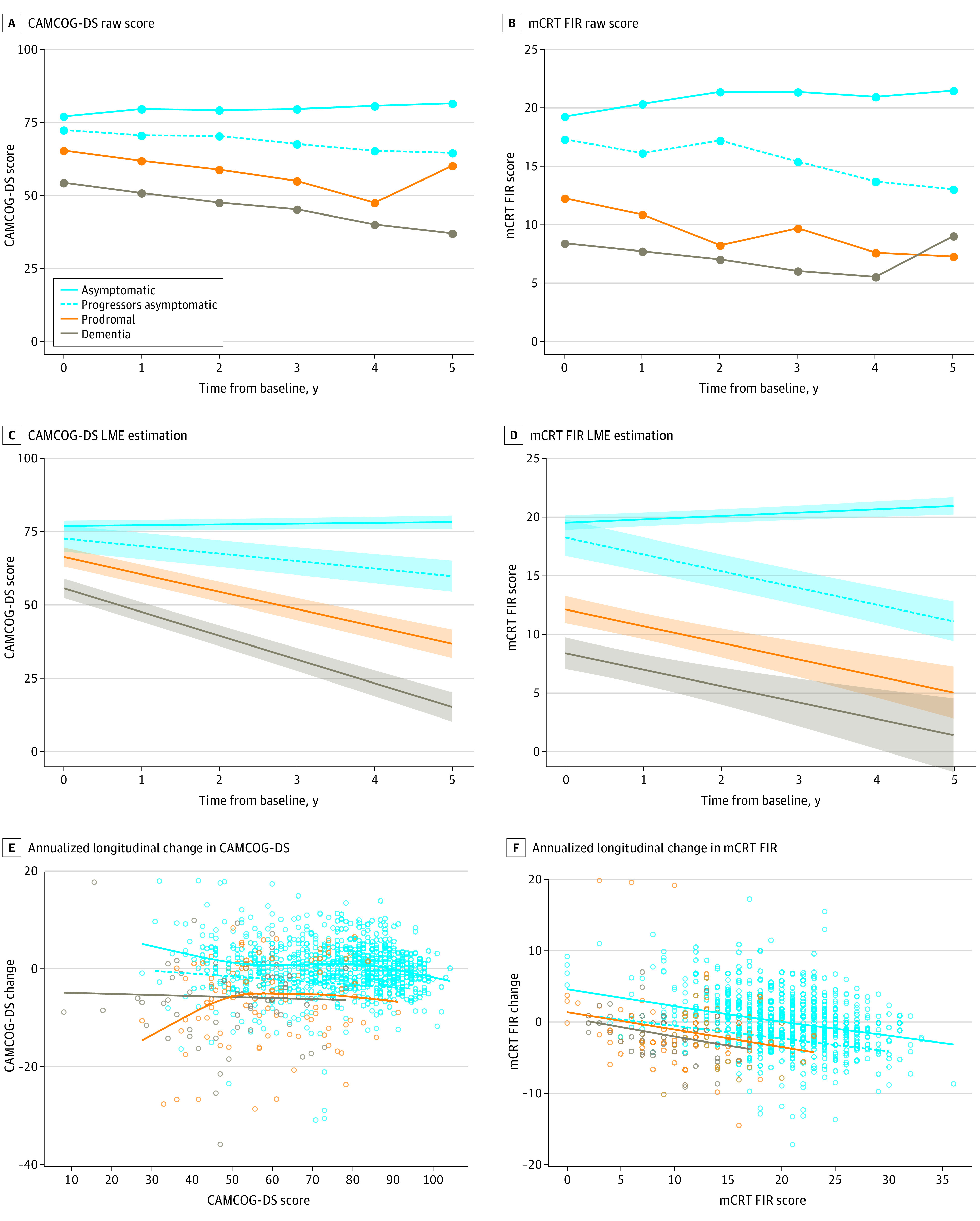
Longitudinal Cognitive Changes by Clinical Diagnosis Showing Floor Effect in the Modified Cued Recall Test (mCRT) Free Immediate Recall (FIR) in Patients With Alzheimer Disease (AD) Dementia Panels A through D show Cambridge Cognitive Examination for Older Adults With Down Syndrome (CAMCOG-DS) (A) and mCRT FIR (B) raw scores and CAMCOG-DS (C) and mCRT FIR (D) linear mixed-effect models estimation along 5 years of follow-up in the different clinical groups and asymptomatic progressors. Panels E and F show that CAMCOG scores continued to decline similarly for all scores (E), but mCRT showed clear floor effects with lower baseline scores associated with less longitudinal decline (F).

Figure 4 shows the longitudinal cognitive changes in the different clinical groups in the combined sample of adults with mild and moderate ID (eFigures 6 and 7 in Supplement show these changes stratified by the level of ID). As expected, there was a progressive decline in CAMCOG-DS and mCRT FIR scores along the AD continuum (for the CAMCOG-DS: asymptomatic individuals vs asymptomatic progressor individuals, mean [SE], −2.00 [0.59] points per year; asymptomatic individuals vs those with prodromal AD, mean [SE], −6.29 [0.59] points per year; asymptomatic individuals vs those with AD dementia, mean [SE], −8.19 [0.71] points per year; for the mCRT: asymptomatic individuals vs asymptomatic progressor individuals, mean [SE], −1.72 [0.17] points per year; asymptomatic individuals vs those with prodromal AD, mean [SE], −1.71 [0.24] points per year; asymptomatic individuals vs those with AD dementia, mean [SE], −1.69 [0.34] points per year; *P* < .001 for all comparisons). However, visual analyses of the trajectories suggested early floor effects for the mCRT FIR in symptomatic individuals and a wider dynamic range for the CAMCOG-DS. To further assess the floor effects (dynamic range in the different groups) of the tests, we plotted the annualized longitudinal change in the score with the baseline performance (Figures 4E and 4F). There was an increasing decline in CAMCOG-DS scores along the AD continuum, but this decline was independent of the baseline scores. However, in the mCRT, although there was a similar decline along the AD continuum, the longitudinal decline was dependent on the baseline scores, and those with scores lower than 10 to 15 did not show longitudinal decline (ie, were at floor effects of the test; see eFigure 8 in the Supplement for the mCRT total immediate recall results). When including the sex to the model, women had a faster cognitive decline than men on the mCRT (mean [SE], 0.11 [0.05] points per year; *P* = .04) but not the CAMCOG-DS; nonetheless, when we stratified by clinical diagnosis, this effect disappeared.

eTables 2 and 3 in the Supplement show the annualized change for the CAMCOG-DS and CRT scores in the different clinical groups and for the different age ranges in asymptomatic individuals. There was significant decline for the CAMCOG-DS after age 45 (mean [SE], −1.25 [0.31] points per year; *P* = .03) and after age 40 for the mCRT FIR (mean [SE], −0.23 [0.13] points per year; *P* = .03).

## Discussion

To our knowledge, this is the largest population-based cohort study of adults with DS with longitudinal clinical and neuropsychological assessments. The large sample size enabled us to estimate for the first time both the risks of progression along the AD continuum at different ages ranges and different follow-up times and the longitudinal cognitive changes by level of ID and by clinical group. We found that, although the level of ID must be considered when using neuropsychological tests for diagnosis, it might not be necessary to monitor longitudinal decline. We also showed for the first time practice effects and floor effects that might impact cognitive end points in clinical trials.

Longitudinal progression along the AD continuum showed a clear age dependency in our study in asymptomatic individuals. Progression was rare before age 40 years but was seen in 57.5% of those older than 50 years after 5 years of follow-up. This age dependency was not seen in patients with prodromal AD, who universally progressed to AD dementia after 5 years. The risk for progression along the AD continuum is very similar to that described in ADAD, now estimated in both populations to be more than 95% in longitudinal studies.^2,5,7^ Data from general population in sporadic AD are more variable, especially because of the study setting and selection criteria (eg, population-based vs convenience cohorts and different mean ages) and different definitions of progression. Petersen^28^ reported an overall annual progression from mild cognitive impairment to AD of 8% to 15% when biomarkers are not evaluated. However, when AD biomarkers are considered, the risk for those with positive biomarkers increases substantially. For example, a previous study^29^ found a 38% (95% CI, 21%-59%) risk of progression from mild cognitive impairment to dementia in those patients with positive amyloid and neurodegeneration biomarkers. Age, as in our study, is another critical factor to consider, especially in cognitively healthy individuals. Progression rates in cognitively healthy euploid individuals increase with age. For example, Roberts et al^30^ found a 1-year risk of progression to mild cognitive impairment of 3.59% in those aged 70 to 74 years, 4.49% in those aged 75 to 79 years, 8.63% in those aged 80 to 84 years, and 13.5% in those aged 85 to 89 years. In short, the main difference between the progression rates and those of the general population are the age at which symptom onset manifest, which is 40 years younger in DS, and the fact that in DS, all patients have (at least) preclinical AD by definition,^2,9^ whereas in the general population the underlying causes of cognitive decline are more heterogeneous.

The cognitive substudy has 4 main findings. First, it confirms the feasibility of performing long-term longitudinal neuropsychological assessments in asymptomatic individuals with DS and in a subset of symptomatic individuals. Second, individuals with mild and moderate ID had similar rates of longitudinal cognitive decline, despite the different offset at all ages. Third, this study found practice effects, most prominently in the episodic memory test. The practice effects obscured the assessment of cognitive decline. Indeed, the observed longitudinal cognitive changes are the net effect of practice effects minus longitudinal cognitive decline. Fourth, we also found floor effects in the episodic memory test, but not in the CAMCOG-DS. The mCRT is, thus, very sensitive to early changes in preclinical and prodromal AD in DS but has clear floor effects (and less applicability) in symptomatic stages to monitor decline. The CAMCOG-DS, although less sensitive to change in preclinical AD, has a better dynamic range in symptomatic individuals and, thus, is better suited for the monitoring of AD progression in symptomatic individuals.

Our findings have several implications for public health and clinical practice. Although the risk of developing dementia before the age of 40 years is low,^31^ cognitive decline (once practice effects are accounted for) starts earlier in individuals with DS (10-15 years before the median diagnosis of prodromal AD),^6,7^ in agreement with previous work^32,33,34^ showing that longitudinal AD-related cognitive decline starts in the fourth decade in people with DS. This temporality of cognitive decline is similar to that described in ADAD, starting with episodic memory decline in the preclinical stage.^2,18,35,36^ Population-based health plans to screen for AD should, therefore, start at approximately age 35 years to detect those individuals at higher risk to progress to dementia. The clinical identification of this high-risk population, most likely in combination with biomarkers, will give people with DS and their families and caregivers the opportunity of an early diagnosis, professional counseling, and treatment.

Our findings also might inform the design of clinical trials. Individuals with DS constitute the largest population of those genetically determined AD and, thus, probably constitute the best population in which to perform preventive clinical trials, even though adults with DS have been largely excluded from AD clinical trials.^2^ Our results underscore this missed opportunity. First, the extremely high progression rates to symptomatic AD confirm that a preventive trial would have high statistical power. Second, we confirm that it is possible to capture and monitor cognitive decline due to AD in this population (in individuals with mild or moderate levels of ID) for the duration of a preventive trial. Importantly, because there are no differences in longitudinal cognitive decline between those with mild and moderate ID, we propose that it might not be necessary to stratify by the level of ID to monitor disease progression in the cognitive end points (as opposed to the use of cross-sectional neuropsychological tests for AD diagnosis),^12^ a result that would undoubtedly facilitate recruitment and power. The practice effects and floor effects must also be considered in the analyses. Practice effects should be considered and modeled, especially when in the context of a trial recruiting participants de novo and from longitudinal cohorts. However, they can increase the dynamic range of the tests and, therefore, their power to detect a response to treatment, especially in the context of short trials. This might explain some of the divergent effects in clinical trials between the cognitive trajectories of the placebo group and historical longitudinal cohorts in sporadic AD and ADAD,^37^ irrespective of the randomization and potential treatment effect.

### Strengths and Limitations

The main strengths of this study are the large sample size and that it comes from a well-characterized large cohort of adults with DS. Thus, we have objective and reliable longitudinal cognitive data obtained with an extensive neuropsychological evaluation.

This study also has limitations. First, it is a single-center study and, thus, needs to be replicated in other cohorts to confirm the generalizability of our results. Second, the follow-up might have been insufficient to fully capture the risk in the younger individuals. Third, individuals with severe and profound ID could not be included in the cognitive analyses. Fourth, we did not analyze the impact of the different biomarkers or *APOE* on progression or cognitive decline and we based all the diagnosis and progression on clinical criteria. Future studies should incorporate biomarkers, especially plasma biomarkers, because of their wider availability and lower costs, to enable better risk stratification of the individuals. We also think that there is a need to develop cognitive tools to assess AD-related cognitive decline in this population suitable for severe or profound ID levels.

## Conclusions

In summary, this study found a very high risk of developing symptomatic AD associated with progressive cognitive decline among adults with DS. These findings support the need for population health plans to screen for AD-related cognitive decline and underscore the imperative and the opportunity to conduct AD preventive clinical trials in adults with DS.

## Notes

Supplement. **eAppendix.** Alzheimer-Down Unit and Clinical Procedures **eFigure 1.** Study Flow Chart **eTable 1.** Cumulative Risk to Clinical AD **eFigure 2.** Clinical Progression by Sex and ID **eFigure 3.** Feasibility of Longitudinal Cognitive Assessments **eFigure 4.** Changes in the mCRT Total Immediate Recall (TIR) With Age by ID **eFigure 5.** Cognitive Trajectories by Age in Asymptomatic DS Showing Learning Effects in Younger Individuals During the First Two Years of Follow-up in the mCRT TIR **eFigure 6.** Longitudinal Cognitive Changes in Mild ID **eFigure 7.** Longitudinal Cognitive Changes in Moderate ID **eFigure 8.** Floor Effects for the mCRT Total Immediate Recall (CRT TIR) **eTable 2.** Annualized Change for the CAMCOG-DS and CRT Scores in the Different Clinical Groups Accounting for Practice Effects in the First Two Years of Follow-up **eTable 3.** Annualized Change for the CAMCOG-DS and CRT Scores in the Different Age Ranges in Asymptomatic Individuals (Progressors and Nonprogressors) Accounting for Practice Effects in the First Two Years of Follow-up
eReferences
