# Supplementary material for: Longitudinal Clinical and Cognitive Changes Along the Alzheimer Disease Continuum in Down Syndrome
Source: JAMA Netw Open. 2022 Aug 5;5(8):e2225573. doi: 10.1001/jamanetworkopen.2022.25573 (PMC9356319; doi:10.1001/jamanetworkopen.2022.25573)
Supplement: Supplement. — eAppendix. Alzheimer-Down Unit and Clinical Procedures eFigure 1. Study Flow Chart eTable 1. Cumulative Risk to Clinical AD eFigure 2. Clinical Progression by Sex and ID eFigure 3. Feasibility of Longitudinal Cognitive Assessments eFigure 4. Changes in the mCRT Total Immediate Recall (TIR) With Age by ID eFigure 5. Cognitive Trajectories by Age in Asymptomatic DS Showing Learning Effects in Younger Individuals During the First Two Years of Follow-up in the mCRT TIR eFigure 6. Longitudinal Cognitive Changes in Mild ID eFigure 7. Longitudinal Cognitive Changes in Moderate ID eFigure 8. Floor Effects for the mCRT Total Immediate Recall (CRT TIR) eTable 2. Annualized Change for the CAMCOG-DS and CRT Scores in the Different Clinical Groups Accounting for Practice Effects in the First Two Years of Follow-up eTable 3. Annualized Change for the CAMCOG-DS and CRT Scores in the Different Age Ranges in Asymptomatic Individuals (Progressors and Nonprogressors) Accounting for Practice Effects in the First Two Years of Follow-up eReferences [file jamanetwopen-e2225573-s001.pdf]

Supplemental Online Content

Videla L, Benejam B, Pegueroles J, et al. Longitudinal clinical and cognitive changes along the Alzheimer disease continuum in Down syndrome. *JAMA Netw Open*. 2022;5(8):e2225573. doi:10.1001/jamanetworkopen.2022.25573

eAppendix. Alzheimer-Down Unit and Clinical Procedures
eFigure 1. Study Flow Chart
eTable 1. Cumulative Risk to Clinical AD
eFigure 2. Clinical Progression by Sex and ID
eFigure 3. Feasibility of Longitudinal Cognitive Assessments
eFigure 4. Changes in the mCRT Total Immediate Recall (TIR) With Age by ID
eFigure 5. Cognitive Trajectories by Age in Asymptomatic DS Showing Learning Effects in Younger Individuals During the First Two Years of Follow-up in the mCRT TIR
eFigure 6. Longitudinal Cognitive Changes in Mild ID
eFigure 7. Longitudinal Cognitive Changes in Moderate ID
eFigure 8. Floor Effects for the mCRT Total Immediate Recall (CRT TIR)
eTable 2. Annualized Change for the CAMCOG-DS and CRT Scores in the Different Clinical Groups Accounting for Practice Effects in the First Two Years of Follow-up
eTable 3. Annualized Change for the CAMCOG-DS and CRT Scores in the Different Age Ranges in Asymptomatic Individuals (Progressors and Nonprogressors) Accounting for Practice Effects in the First Two Years of Follow-up
eReferences

This supplemental material has been provided by the authors to give readers additional information about their work.

© 2022 Videla L et al. *JAMA Network Open.*

eAppendix: Alzheimer-Down Unit and Clinical Procedures

Down syndrome is now considered a genetically determined form of AD[4]. The premorbid intellectual disability associated with Down syndrome complicates the diagnosis as it can overshadow AD-related cognitive decline and explains the floor effects in traditional neuropsychological tests in most individuals with DS. This population requires adapted tests to assess cognitive performance,[1] which can overshadow AD-related cognitive decline and requires adapted tests. Furthermore, health professional from general population do not feel confident when attending people with DS.[4]

The Alzheimer-Down Unit was founded in 2014 and it was recognized by the Catalan government as the reference center for neurological pathologies for people with DS from all around Catalunya. The main aim of the Unit is the prevention, early detection and treatment of AD in adults with Down syndrome and we developed a pioneer health plan for adults with DS unique worldwide including annual medical and neuropsychological assessments. The Alzheimer-Down Unit is composed by a multidisciplinary team including neurologist, neuropsychologists, nurses, social workers and administrative staff, specialized in both, intellectual disabilities and neurodegenerative disorders.

- <u>Recruitment and retention</u>: It is estimated that in Catalonia there are 3,500 people with Down syndrome. We are the medical reference center for adults with DS and we have evaluated and attended almost 1,000 people with DS. Medical care is free and the sample is representative of the population with DS in Catalonia. This health plan has been developed in conjunction with the Fundació Catalana Síndrome de Down and we have disseminated it in different foundations, residences, occupational centers, special employment centers, etc to expand recruitment. For those patients who were not able to physically attend to our center, we developed the Domiciliary Alzheimer Visiting in Down Syndrome (DAVIS). This program allowed us to go to different centers in the Catalan territory to attend individuals with difficulties to move to Barcelona and perform our complete neurological and neuropsychological assessments.

© 2022 Videla L et al. *JAMA Network Open.*

Another strategy to control a potential selection bias was facilitating telemedicine medical visits (not cognitive) for those patients with advanced dementia or severe medial problems that could no longer attend our center.

Regarding retention strategies, we annually contact participants to schedule and/or remind the visits, we also disclose all tests results to the participants and work closely with the primary care physicians. Furthermore, we do scientific dissemination through periodic newsletters and conferences to families.

- Medical visit: Our routine medical/neurological visit consists in a structured anamnesis with the patient and his caregiver and a physical examination performed by expert neurologists including the Cambridge Examination for Mental Disorders of the Elderly with Down Syndrome and Others with Disabilities Intellectual (CAMDEX-DS) interview and the Neuropsychiatric Inventory (NPI). We also collect demographic data, clinical and neurological history including epilepsy associated risk factors, detailed semiology of epileptic seizures and treatments. We finally offer (and recommend) to perform annual general blood test.

- Cognitive assessment: Cognitive test commonly used in general population, as MMSE or the FCSRT, scores at floor levels in people with ID and are useless to distinguish between premorbid ID cognitive impairment and AD-related cognitive decline. For this reason, it is recommended to use adapted tests specially designed for people with ID to avoid floor effects. Following this recommendation, our neuropsychological protocol includes the Cambridge Cognitive Examination for Older Adults with Down's Syndrome (CAMCOG-DS) Spanish version[5], the modified Cued Recal Test (mCRT)[6] and the Cats&Dogs test and a cancellation task. At baseline, we also establish the individuals' intellectual disability level following the Diagnostic and Statistical Manual of Mental Disorders, Fifth Edition (DSM-V) criteria and we obtain the Intellectual Quotient (IQ) assessed with the Kaufman Brief Intelligence Test Spanish version[7], to further support the ID level classification. However, when there is suspicion of cognitive decline the IQ is not obtained as it may be biased.

© 2022 Videla L et al. *JAMA Network Open.*

- Diagnostic process: After this two independent visits, neurologists and neuropsychologists clinically classify the subject, blind to the information collected in the parallel visit, to avoid circularity in our data. After this independent diagnosis, there is a consensus meeting between both professionals to determine the final diagnose. If there is an agreement, the diagnosis is considered as definitive. Otherwise, the case is reviewed together with the complementary test if available and with other team members if needed, to reach a consensus in the definitive diagnosis.

The clinical classification, which is made at a consensus conference between the neurologist and the neuropsychologist, includes four different groups: 1) Asymptomatic: when there is no clinical or neuropsychological suspicion of AD; 2) Prodromal AD: when there is suspicion of AD, but symptoms do not fulfill criteria for dementia; 3) AD dementia: when there is full blown AD dementia with cognitive decline and impairment in activities of daily living; 4) Uncertain or non-degenerative neurocognitive disorder: when the person has a medical, pharmacological or psychiatric condition interfering with cognition or activities of daily living, but no suspicion of neurodegenerative origin. Of note, in some instances these conditions are treatable and reversible and individuals can change their clinical classification to one of the other three categories. We consider progression along the AD continuum when there are clinical changes coherent with AD diagnosis and no other medical or socio-emotional conditions justifying the changes. We consider progression from asymptomatic to prodromal AD when there are relevant cognitive or behavioral changes but there is no interference in daily living activities, and from prodromal to AD dementia when the cognitive and behavioral changes do interfere in personal autonomy of patients.

© 2022 Videla L et al. *JAMA Network Open.*

eFigure 1: Study Flow Chart

In this study we included 632 adults with Down syndrome (DS), male or female, over 18 years old from the population-based health plan at the Alzheimer-Down Unit in Barcelona, Spain. This plan was designed to screen Alzheimer's disease (AD) dementia and other neurological pathology related with age in this population.[1,2]

Inclusion criteria were: 1) having Down syndrome; 2) ≥18 years old; 3) at least 2 clinical visits. For the cognitive analyses we excluded all those individuals with severe, profound or unknown levels of intellectual disability as these individuals perform at floor levels even when using adapted tests.[3] We also excluded those individuals who did not complete the neuropsychological assessment due to different causes (for details, see efigure1).

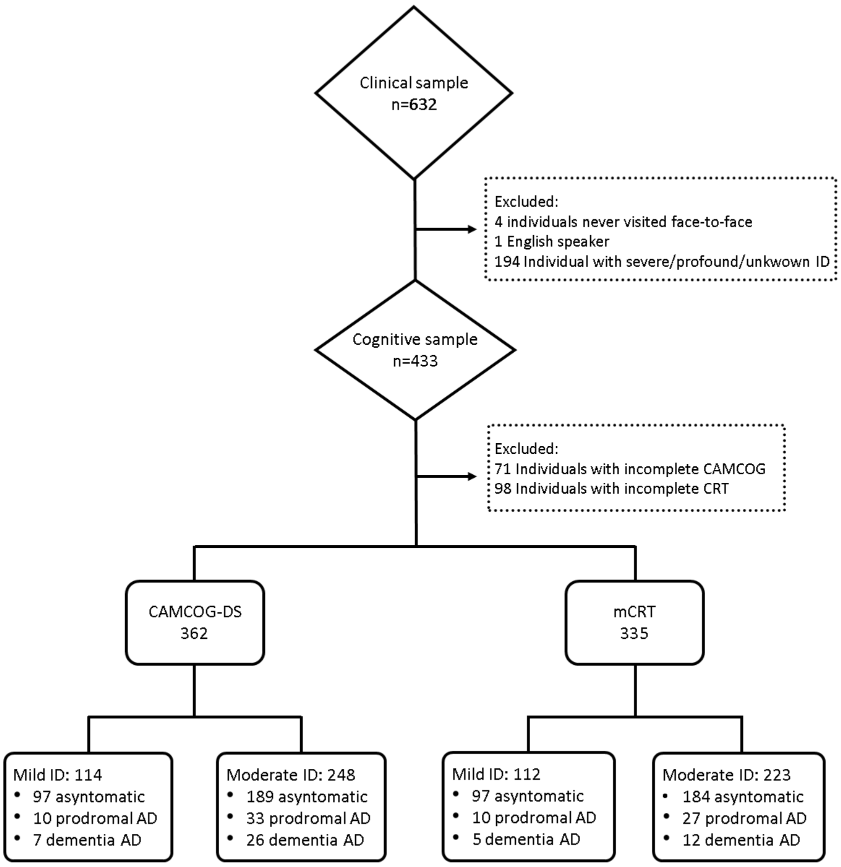

eFigure 1: Study flow chart.

Abbreviations: ID, Intellectual Disability CAMCOG-DS, Cambridge Cognitive Examination for Older Adults with Down's Syndrome; mCRT FIR, Free immediate Recall score of the modified Cued Recall Test; AD, Alzheimer's disease

© 2022 Videla L et al. *JAMA Network Open.*

eTable 1: Cumulative Risk to Clinical AD

As explained in the main text, we estimated the clinical progression rates along Alzheimer's disease continuum in the whole sample and for the different age-ranges in asymptomatic and prodromal AD individuals separately. Overall, after 5 years of follow-up, 17.1% of the asymptomatic individuals had progressed to symptomatic AD and 94.1% of the prodromal group had progressed to dementia. eTable 1 shows the cumulative progression risk at different follow-up times (years 1, 3 and 5) in the whole cohort, as well as stratified by

age-ranges and clinical diagnosis

eTable 1: Progression rates of asymptomatic and prodromal AD at different follow-up time points (years 1, 3 and 5) in the whole cohort and in the different age-ranges

	Asymptomatic			Prodromal AD		
	Risk estimates Percentage (95% CI)			Risk estimates Percentage (95% CI)		
	Year 1	Year 3	Year 5	Year 1	Year 3	Year 5
Age group						
ALL	1.4 (0.3-2.6)	9.4 (6.2-12.5)	17.1 (12.5-21.5)	31.1 (18.7-41.5)	74.6 (60.3-83.8)	94.1 (82.6-98.0)
<40	0.0 (0.0-0.0)	0.6 (0.0-1.8)	0.6 (0.0-1.8)	NA	NA	NA
40-44	2.7 (0.0-6.3)	8.3 (0.9-15.1)	21.2 (8.0-32.5)	60.0 (0.0-86.3)	80.0 (0.0-96.5)	NA
45-49	0.0 (0.0-0.0)	23.5 (10.2-34.8)	41.3 (23.1-55.3)	39.9 (15.8-57.2)	80.3 (48.3-92.5)	93.4 (57.5-99.0)
>50	9.6 (1.2-17.3)	36.7 (20.9-49.3)	57.5 (38.2-70.8)	28.1 (11.6-41.5)	77.7 (57.4-88.3)	96.8 (78.1-99.5)

Abbreviations: AD, Alzheimer's disease; 95% CI, 95% Confidence interval; NA, Not applicable

© 2022 Videla L et al. *JAMA Network Open.*

eFigure 2: Clinical Progression by Sex and ID

eFigure 2 shows the Kaplan-Meier curves for the clinical progression in in asymptomatic and prodromal AD individuals separately, stratified by sex (panels A and B), as well as, by level of intellectual disability (panels C and D). Women in the prodromal stage of the disease progressed faster than men (*P*= .039), whereas no statistical differences were found in the asymptomatic sample (*P*= .11). We found no differences when accounting by intellectual disability level.

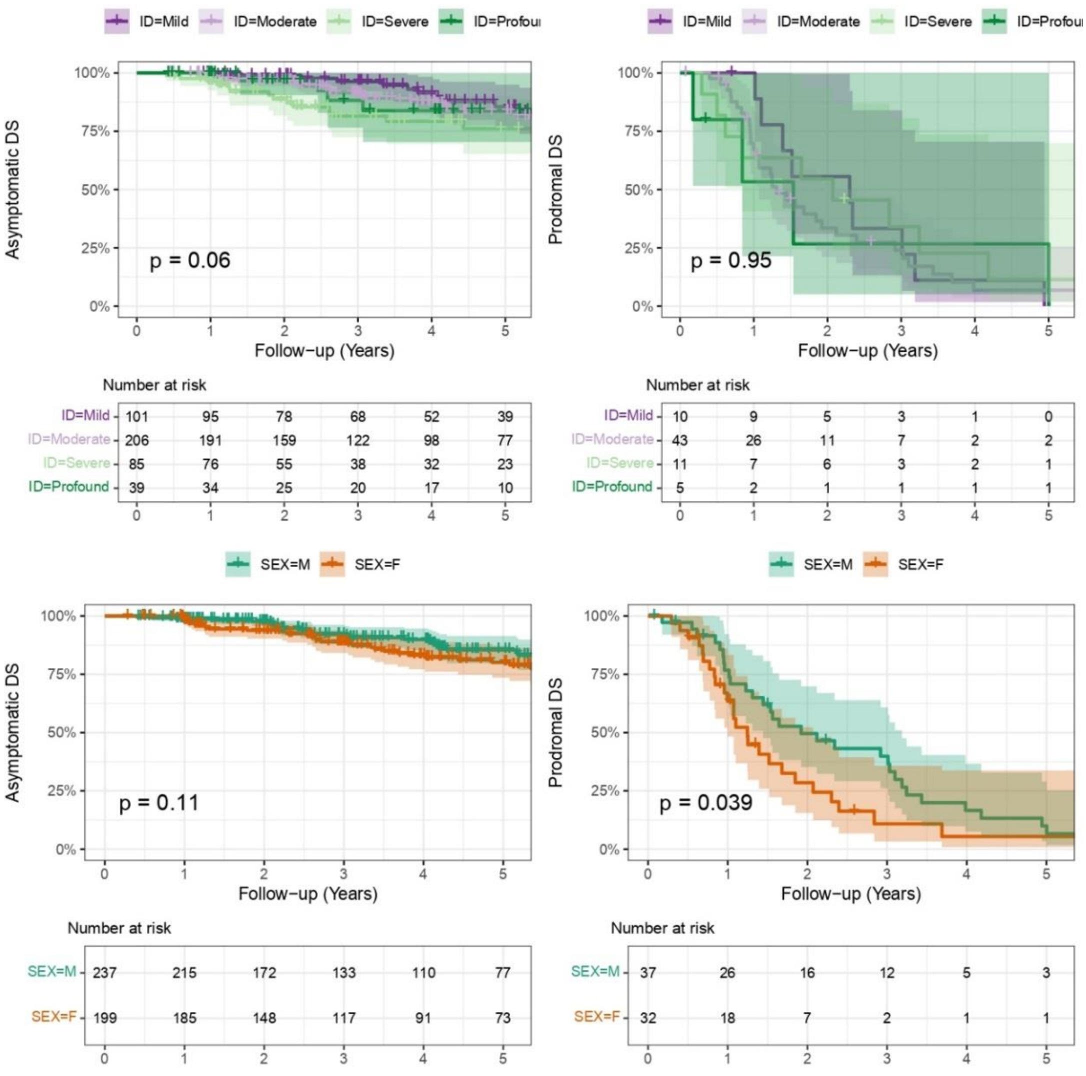

eFigure 2: Clinical progression rate stratified by sex (panels A and B) and by intellectual disability: mild, moderate, severe and profound (panels C and D), in asymptomatic individuals (left panels) and in prodromal AD (right panels).

Abbreviations: DS, Down syndrome; F, Female; M, Male.

© 2022 Videla L et al. *JAMA Network Open.*

eFigure 3: Feasibility of Longitudinal Cognitive Assessments

For the longitudinal cognitive analyses, we studied the percentage of individuals who were able to complete the CAMCOG-DS and the mCRT at the different follow-ups by clinical diagnosis and level of ID (eFigure 3). Most asymptomatic participants were able to complete both tests at baseline (95.9% [328/342] for CAMCOG-DS and 94.7% [324/342] for mCRT), but the completion rates were lower in individuals with prodromal AD and AD dementia (87.1% [74/85] and 71.6% [66/85] for CAMCOG-DS and 77.7% [53/74] and 43.2% [32/74] for the mCRT, respectively).

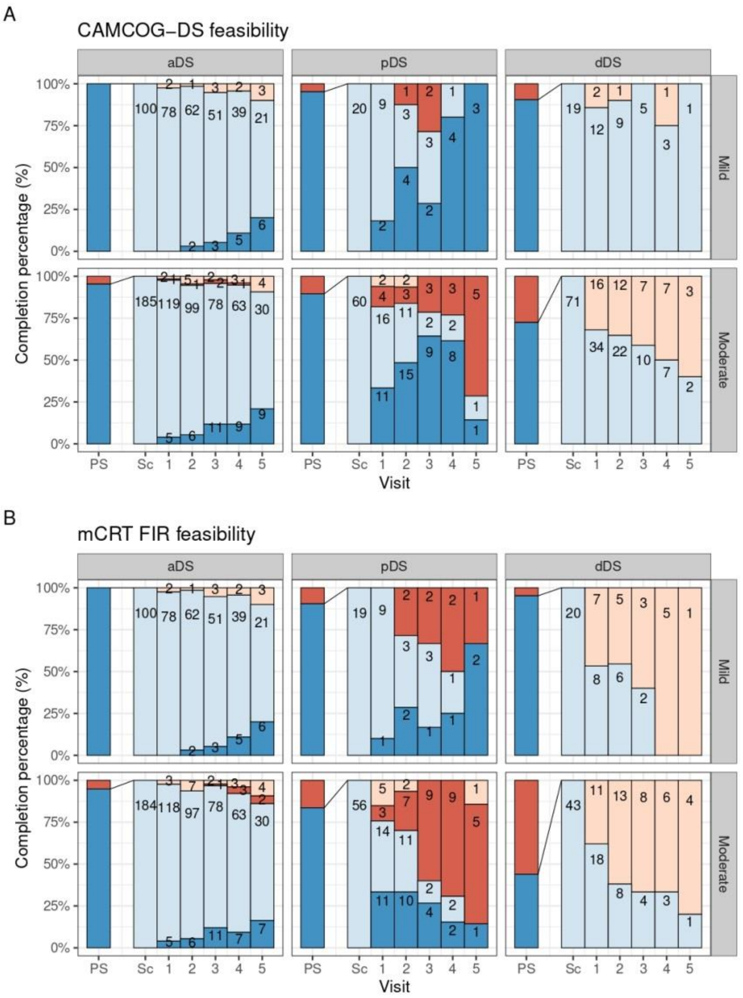

eFigure 3: Longitudinal
completion rates for the CAMCOG-DS and mCRT by level of intellectual disability and by diagnostic group. PS bar represents feasibility at baseline, importantly, only those individuals completing assessment at PS are represented in the following histograms. Dark blue represents subjects completing the test. Red color represents subjects unable to complete the test. Bright colors represent those subjects progressing along Alzheimer's disease continuum and dull colors represents subjects who remained in the same clinical category along the whole follow-up.

Abbreviations: aDS, asymptomatic Down Syndrome; pDS, prodromal Down syndrome; dDS, Dementia Down syndrome; PS, PreScreening; Sc, Screening;

© 2022 Videla L et al. *JAMA Network Open.*

eFigure 4: Changes in the mCRT Total Immediate Recall (TIR) With Age by ID.

As found in CAMCOG-DS and mCRT FIR (shown in the main text), the mCRT TIR scores were higher in individuals with mild ID at all ages (P < .001) (upper row), but the interaction term ID*age was not significant, suggesting that there were no differences in the trajectories between the ID groups. Similarly, the annualized change did not differ between individuals with mild and moderate ID (lower row), showing that despite baseline differences (off-sets), individuals with mild and moderate ID showed similar cognitive decline with age (eFigure 4).

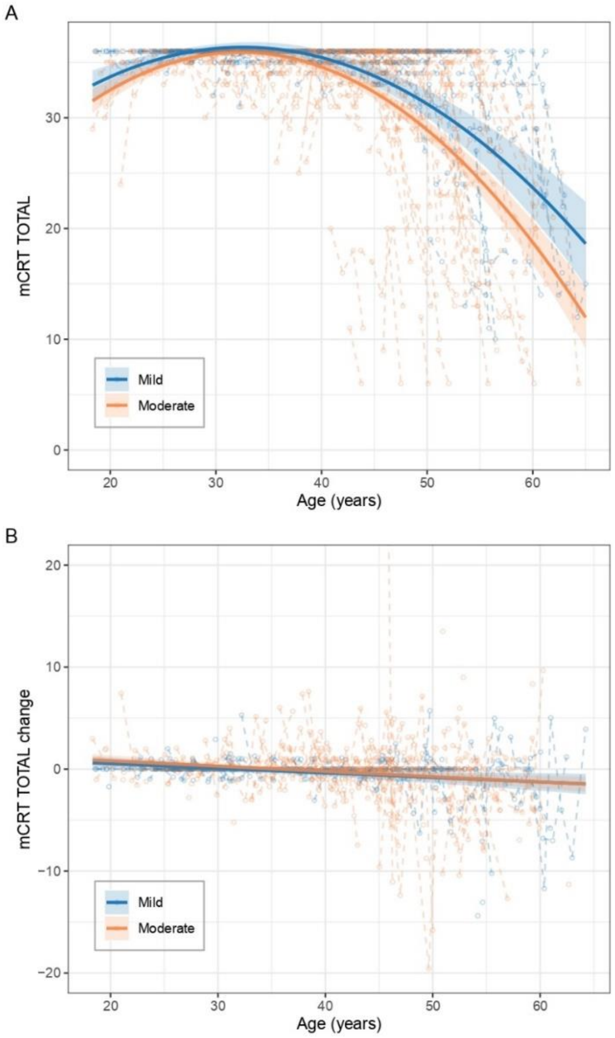

eFigure 4 shows changes in the mCRT TIR scores with age in individuals with mild and moderate levels of ID separately. Quadratic relation between age mCRT TIR raw scores in mild (blue dots) and moderate (orange dots) ID. Lower figures show the relation between the annualized cognitive change and age by ID mCRT TIR.

Abbreviations: mCRT TIR, Total Immediate Recall score of the modified Cued Recall Test; ID, intellectual disability.

© 2022 Videla L et al. *JAMA Network Open.*

eFigure 5: **Cognitive Trajectories by Age in Asymptomatic DS Showing Learning Effects in Younger Individuals During the First Two Years of Follow-up in the mCRT TIR.**

eFigure 5 shows the presence of practice effects with the mCRT TIR. We found similar results to the CAMCOG-DS and the mCRT FIR, but limited due to a higher quantity of ceil scores. Again, these analyses showed practice effects during the first two years in asymptomatic individuals, either as early increases in the cognitive test, with subsequent stabilization, or with subsequent decline (top row). The middle panel shows the estimated trajectories for the mCRT TIR scores for the first two years of follow-up and those beyond separately to truly estimate the cognitive decline when there are no practice effects (those after two years of follow-up). We finally estimated the longitudinal trajectory of change in the different age ranges (lower panel). Practice effects (apparent as longitudinal improvement in the cognitive tests) were clear in younger individuals.

© 2022 Videla L et al. *JAMA Network Open.*

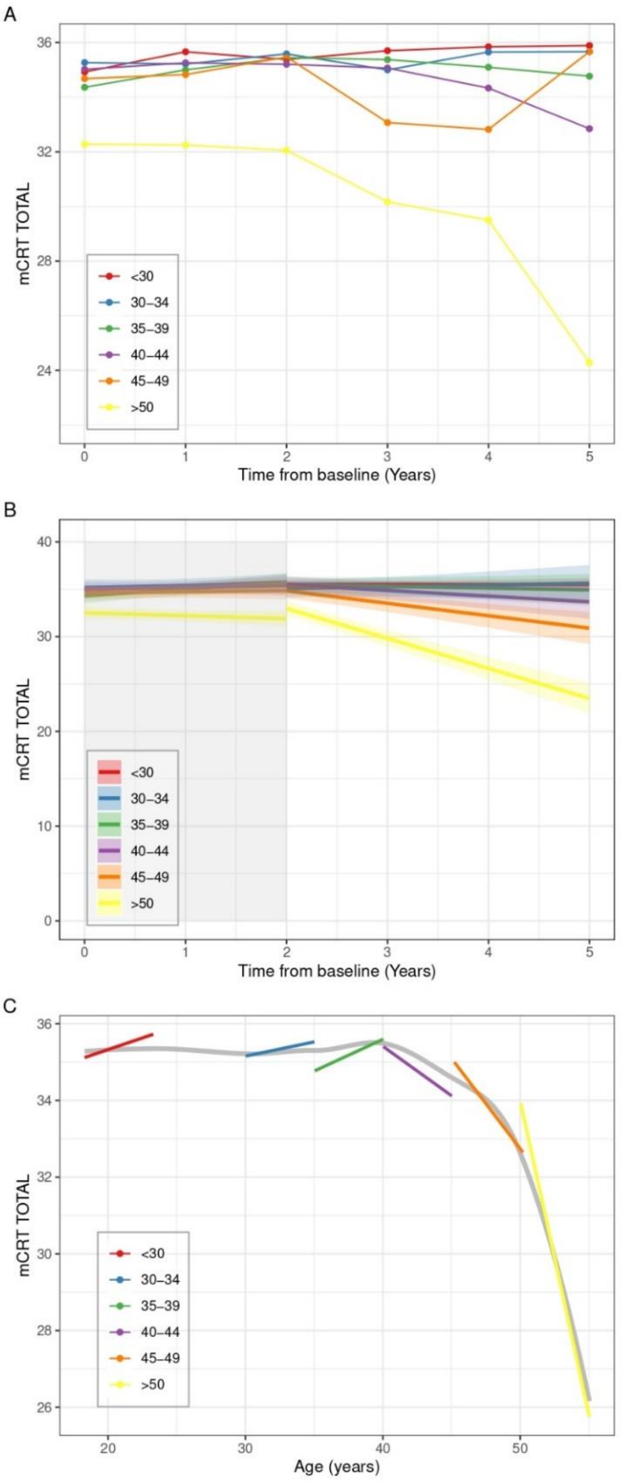

eFigure 5 shows the cognitive trajectories by age ranges in asymptomatic DS showing learning effects in younger individuals during the first two years of follow-up. mCRT TIR raw scores by age ranges along 5 years of follow-up (A). mCRT TIR estimated slopes for the cognitive trajectories during the first two years of follow-up and beyond calculated separately by age ranges during the follow-up (B). Finally, mCRT TIR practice effects were seen several years after a decline in (baseline) cognitive scores with age was observed. Light green represents younger individuals and dark green the oldest. (C)

Abbreviations: DS, Down syndrome; mCRT TIR, Total Immediate Recall score of the modified Cued Recall Test.

© 2022 Videla L et al. *JAMA Network Open.*

eFigure 6: Longitudinal Cognitive Changes in Mild ID

eFigure 7: Longitudinal Cognitive Changes in Moderate ID

eFigures 6 and 7 show the longitudinal cognitive changes in the different clinical groups stratified by the level of ID (mild [eFigure 6] and moderate [eFigure 7] separately) to further show that the longitudinal cognitive evolution of both groups was similar. As expected, there was a progressive decline in CAMCOG-DS and mCRT FIR scores along the AD continuum in both groups.

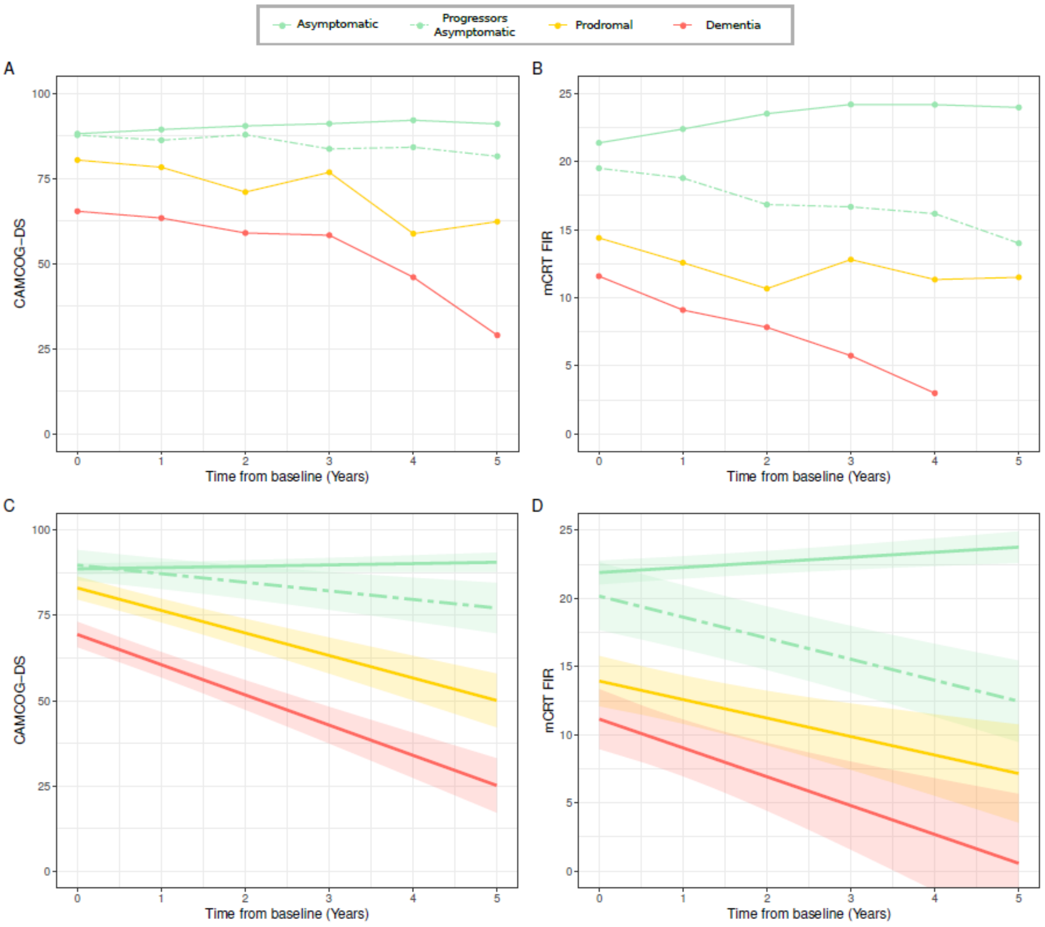

eFigure 6: Longitudinal cognitive changes by clinical diagnose in individuals with a mild intellectual disability. The upper panels represent the CAMCOG-DS (A) and the mCRT FIR (B) raw scores along 5 years of follow-up in the different clinical diagnoses and in aDS-progressors. The bottom panels represent the CAMCOG-DS (C) and mCRT FIR (D) scores LME estimation along 5 years of follow-up in the different clinical groups and aDS-progressors. Asymptomatic DS individuals are represented in the green line, asymptomatic progressors are represented in the green dotted line, patients with prodromal AD and AD dementia are represented in yellow and red lines, respectively

Abbreviations: mCRT, modified Cued Recall Test; CAMCOG-DS, Cambridge Cognitive Examination for Older Adults with Down 's syndrome.

© 2022 Videla L et al. *JAMA Network Open.*

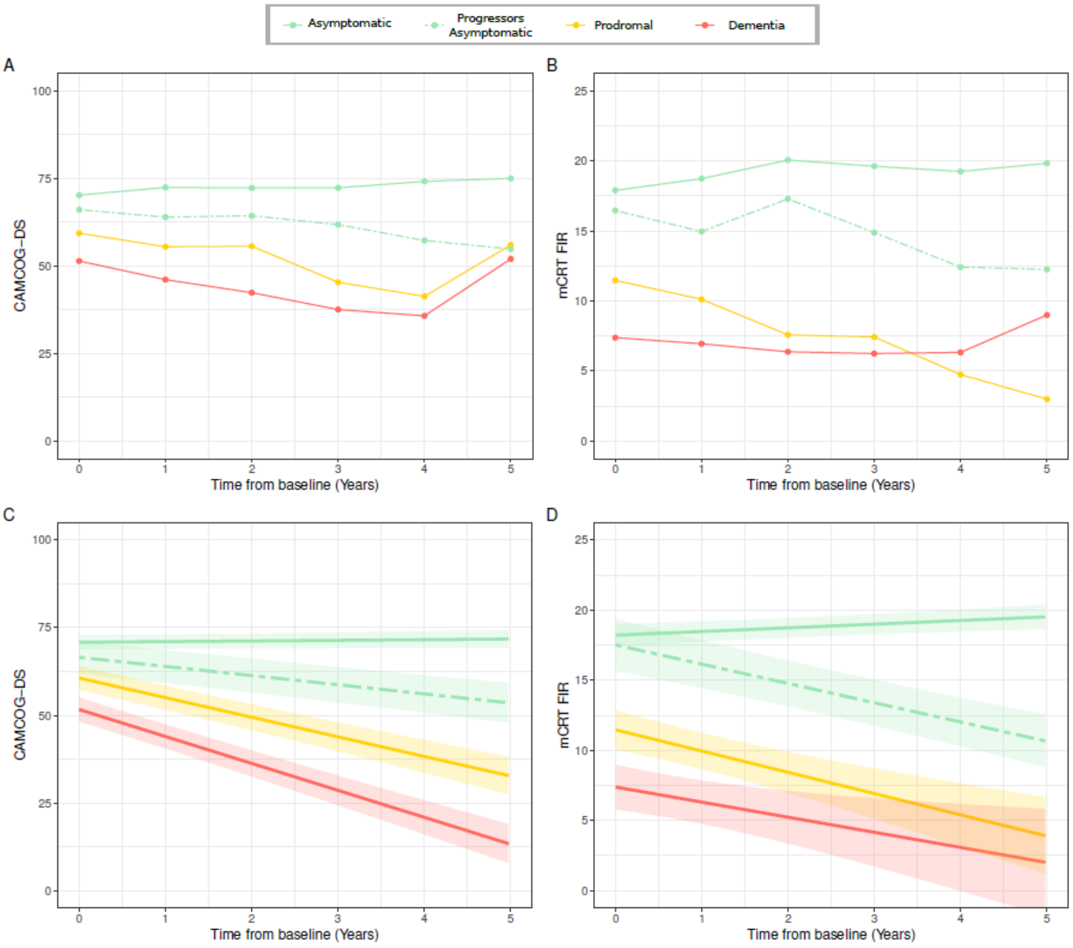

eFigure 7: Longitudinal cognitive changes by clinical diagnose in individuals with a <u>moderate intellectual disability</u>. The upper panels represent the CAMCOG-DS (A) and the mCRT FIR (B) raw scores along 5 years of follow-up in the different clinical diagnoses and in aDS-progressors. The bottom panels represent the CAMCOG-DS (C) and mCRT FIR (D) scores LME estimation along 5 years of follow-up in the different clinical groups and aDS-progressors. Asymptomatic DS individuals are represented in the green line, asymptomatic progressors are represented in the green dotted line, and patients with prodromal AD and AD dementia are represented in yellow and red lines, respectively

Abbreviations: mCRT, modified Cued Recall Test; CAMCOG-DS, Cambridge Cognitive Examination for Older Adults with Down's syndrome.

© 2022 Videla L et al. *JAMA Network Open.*

eFigure 8: Floor Effects for the mCRT Total Immediate Recall (CRT TIR)

eFigure 8 shows the longitudinal cognitive changes in the different clinical groups in the combined sample of adults with mild and moderate ID in the mCRT TIR scores. As expected, there was a progressive decline in the mCRT TIR scores along the AD continuum ($P < .001$ for all comparisons vs asymptomatic non-progressors). However, visual analyses of the trajectories suggested early floor effects for the mCRT TIR in symptomatic individuals (same as mCRT FIR shown in the main text). To further assess the floor effects (dynamic range in the different groups) of the tests, we plotted the annualized longitudinal change in the score with the baseline performance (supplementary Figure 8, lower panel). In the mCRT TIR, although there was a similar increasing decline along the AD continuum, the longitudinal decline was dependent of the baseline scores, and those with scores lower did not show longitudinal decline (were at floor effects of the test).

© 2022 Videla L et al. *JAMA Network Open.*

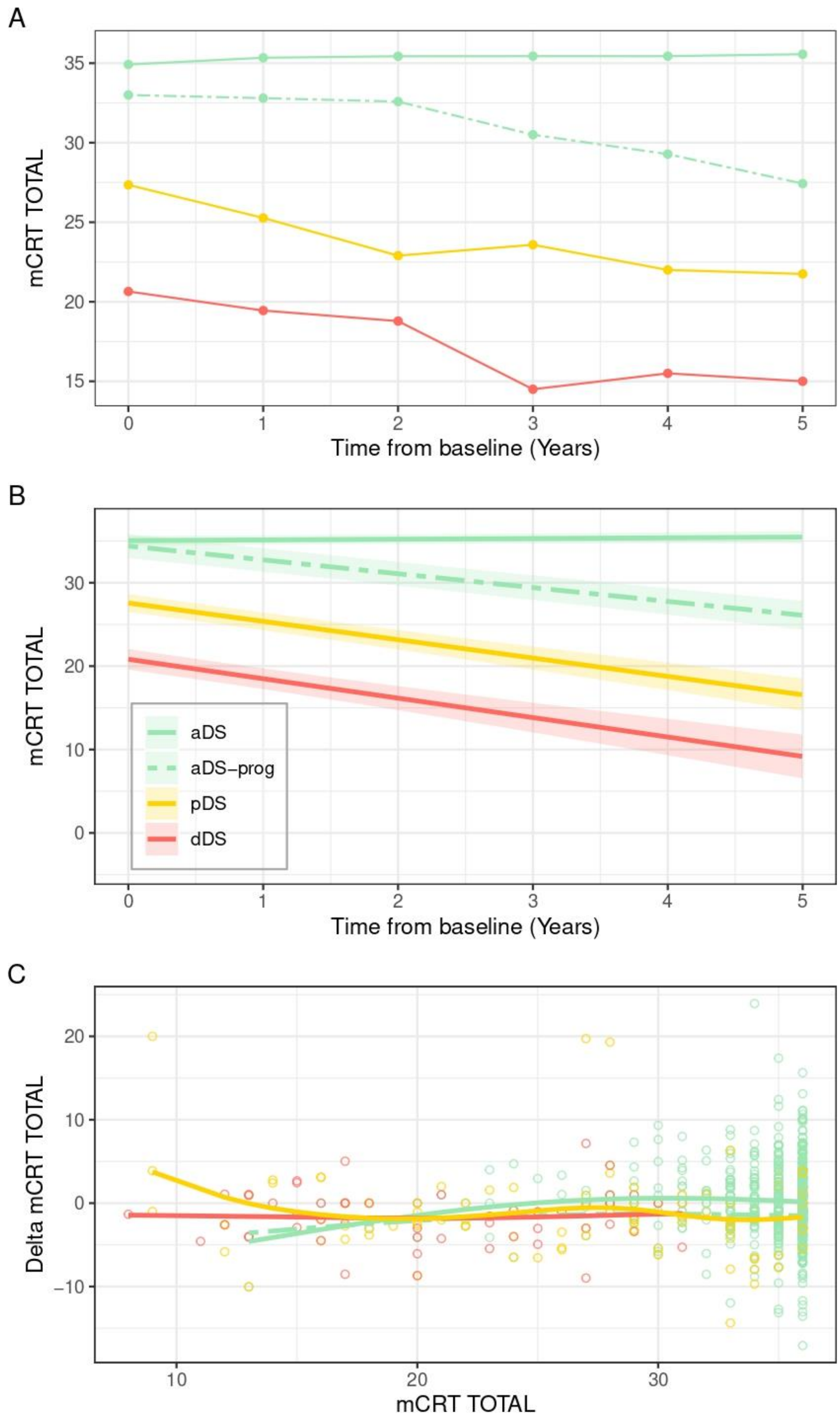

eFigure 8 shows the longitudinal cognitive changes by clinical diagnosis show floor effect in the mCRT TIR in patients with AD dementia. First, the mCRT TIR raw scores (A) and the mCRT TIR (B) LME estimation along 5 years of follow-up in the different clinical groups and asymptomatic-progressors. mCRT TIR showed clear floor effects with lower baseline scores associated with less longitudinal decline in individuals with dementia. Asymptomatic DS represented in the green line, asymptomatic progressors represented in the green dotted line, prodromal DS in yellow and demented DS in red.

Abbreviations: AD, Alzheimer´s disease; mCRT TIR, Total Immediate Recall score of the modified Cued Recall Test; LME, Linear Mixed Effect Models; DS, Down syndrome.

© 2022 Videla L et al. *JAMA Network Open.*

eTables 2 and 3: Annualized cognitive change

eTable 2: Annualized Change for the CAMCOG-DS and CRT Ccores in the Different Clinical Groups Accounting for Practice Effects in the First Two Years of Follow-up

eTable 3: Annualized Change for the CAMCOG-DS and CRT Scores in the Different Age Ranges in Asymptomatic Individuals (Progressors and Nonprogressors) Accounting for Practice Effects in the First Two Years of Follow-up

Supplementary tables 2 and 3 show the annualized change for the CAMCOG-DS and CRT scores in the different clinical groups and for the different age ranges in asymptomatic individuals.

	**CAMCOG** Annualized change			**CRT** Annualized change		
	Whole FU	FU <2y	FU >2y	Whole FU	FU <2y	FU >2y
Asymptomatic	-0,2	1,3	-0,7	0,0	0,8	-0,4
Asymptomatic non-progressors	0,3	1,6	0,0	0,3	1,0	0,0
Asymptomatic progressors	-2,6	-1,0	-3,6	-1,4	-0,6	-2,3
Prodromal AD	-5,9	-3,2	-9,9	-1,4	-1,8	-1,2
AD Dementia	-8,1	-7,8	-10,4	-1,4	-1,3	-0,7

eTable 2: Annualized Change for the CAMCOG-DS and CRT Ccores in the Different Clinical Groups Accounting for Practice Effects in the First Two Years of Follow-up.

Abbreviations: mCRT, modified Cued Recall Test; CAMCOG-DS, Cambridge Cognitive Examination for Older Adults with Down's syndrome.

<table>
<tr><th colspan="7">Asymptomatic</th></tr>
<tr><th rowspan="2">(All |non-progressors |progressors)</th><th colspan="3">CAMCOG
Annualized change</th><th colspan="3">CRT
Annualized change</th></tr>
<tr><th>Whole FU</th><th>FU <2y</th><th>FU >2y</th><th>Whole FU</th><th>FU <2y</th><th>FU >2y</th></tr>
<tr><td><30</td><td>0.5|0.5|NA</td><td>1.6|1.6|NA</td><td>0.3|0.3|NA</td><td>0.4|0.4|NA</td><td>1.7|1.7| NA</td><td>0.1|0.0 NA</td></tr>
<tr><td>30-34</td><td>0.3|0.4|NA</td><td>1.2|1.2|NA</td><td>0.2|0.2|NA</td><td>0.2|0.2| NA</td><td>1.1|1.1| NA</td><td>0.4|0.4| NA</td></tr>
<tr><td>35-39</td><td>-0.1|-0.1|-2.2</td><td>1.8|1.8|2.2</td><td>-0.2|-0.2|1.9</td><td>0.3|0.3|5.2</td><td>0.7|0.6|4.3</td><td>-0.4|-0.3|-7.1</td></tr>
<tr><td>40-44</td><td>-0.3|0.1|-2.1</td><td>1.4|1.5|0.4</td><td>-0.7|-0.2|-2.2</td><td>-0.2|0.1|-1.9</td><td>0.3|0.4|0.1</td><td>-0.8|-0.2|-2.9</td></tr>
<tr><td>45-49</td><td>-1.3|0.2|-2.6</td><td>0.3|2.3|-1.7</td><td>-2.4|-0.6|-4.2</td><td>-0.3|0.3|-1.0</td><td>0.8|0.1|0.3</td><td>-0.6|0.0|-1.3</td></tr>
<tr><td>>50</td><td>-2.3|0.8|-2.8</td><td>-0.5|1.5|-1.3</td><td>-3.4|0.7|-4.1</td><td>-1.3|-0.1|-1.5</td><td>-0.8|0.8|-1.4</td><td>-2.1|0.4|2.3</td></tr>
</table>

eTable 3: Annualized Change for the CAMCOG-DS and CRT Scores in the Different Age Ranges in Asymptomatic Individuals (Progressors and Nonprogressors) Accounting for Practice Effects in the First Two Years of Follow-up.

Abbreviations: mCRT. modified Cued Recall Test; CAMCOG-DS. Cambridge Cognitive Examination for Older Adults with Down's syndrome.

© 2022 Videla L et al. *JAMA Network Open.*

eReferences

1. Blesa R, Trias C, Fortea J, Videla S. Alzheimer ’ s disease in adults with Down syndrome : a challenge. *T21RS Sci Soc Bull 2015*. 2015;2015(2).

2. Fortea J, Zaman SH, Hartley S, Rafii MS, Head E, Carmona-Iragui M. Alzheimer’s disease associated with Down syndrome: a genetic form of dementia. *Lancet Neurol*. 2021;20(11):930-942. doi:10.1016/S1474-4422(21)00245-3

3. Benejam B, Videla L, Vilaplana E, et al. Diagnosis of prodromal and Alzheimer’s disease dementia in adults with Down syndrome using neuropsychological tests. *Alzheimer’s Dement Diagnosis, Assess Dis Monit*. 2020;12(1). doi:10.1002/dad2.12047

4. Strydom A, Livingston G, King M, Hassiotis A. Prevalence of dementia in intellectual disability using different diagnostic criteria. *Br J Psychiatry*. 2007;191(AUG.):150-157. doi:10.1192/bjp.bp.106.028845

5. Esteba-Castillo S, Dalmau-Bueno A, Ribas-Vidal N, Vilà-Alsina M, Novell-Alsina R, García-Alba J. Adaptation and validation of CAMDEX-DS (Cambridge Examination for Mental Disorders of Older People with Down’s Syndrome and Others with Intellectual Disabilities) in Spanish population with intellectual disabilities. *Rev Neurol*. 2013;57(8):337-346. doi:10.33588/rn.5708.2013259

6. Devenny DA, Zimmerli EJ, Kittler P, Krinsky-McHale SJ. Cued recall in early-stage dementia in adults with Down’s syndrome. *J Intellect Disabil Res*. 2002;46(6):472-483. doi:10.1046/j.1365-2788.2002.00417.x

7. Kaufman AS KN. *Kaufmann Brief Intelligence Test*. 3 edition. (Ediciones T, ed.). Madrid; 2004.

© 2022 Videla L et al. *JAMA Network Open.*
